# Stem cell transplantation in strategies for curing HIV/AIDS

**DOI:** 10.1186/s12981-016-0114-y

**Published:** 2016-09-13

**Authors:** Gero Hütter

**Affiliations:** 1 Cellex CC, Fiedlerstr. 36, 01307 Dresden, Germany; 2 Universitätsklinikum Carl Gustav Carus der TU Dresden, Dresden, Germany

**Keywords:** HIV-1, Stem cell transplantation, Gene therapy, Cell therapy, Adoptive cell transfer

## Abstract

HIV-1 can persist in a latent form in resting memory CD4+ cells and macrophages carrying an integrated copy of the HIV genome. Because of the presence of these stable reservoir cells, eradication by antiretroviral therapy is unlikely and in order to achieve eradication, alternative treatment options are required. Stem cell transplantation has been considered previously to effect the clinical course of HIV-infection but in practice eradication or virus control was not achievable. However, modifications of stem cell transplantation using natural or artificial resistant cell sources, combination with new techniques of gene editing or generating cytotoxic anti HIV effector cells have stimulated this field of HIV cell therapy substantially. Here, we look back on 30 years of stem cell therapy in HIV patients and discuss most recent developments in this direction.

## Background

The infection with human immunodeficiency virus (HIV) leads to a lifelong infection. Without antiretroviral treatment (ART) a considerable part of infected people die from the acquired immunodeficiency syndrome (AIDS) caused by evolving loss of T-helper cells. With ART the replication of HIV can be effectively suppressed in the peripheral blood and the number of functional T-helper cells returns back to normal or close to normal levels. Because of the persistent infection ART has to be continued during the whole life-span of the patients. Immunological effects of the chronic viral inflammation and long-time side effects of the medication lead to consecutive secondary complications affecting quality of live and survival in HIV+ patients [1].

Because HIV replication is mainly limited to immune cells (mainly T-lymphocytes and macrophages) it is not fallacious to call the HIV a hematological infection. Therefore, from the earliest days of the appearance of HIV as major worldwide health problem, speculations on the impact of hematological treatment strategies to benefit or even cure HIV infection have evolved during the past decades [2].

Developments in oncology like stem cell transplantation, adoptive cell therapy, gene therapy, tumor vaccination, or epigenetics, etc. have been also an inspiration for new strategies in HIV treatment and especially for ambitious tasks like eradication and cure. Since the early 1980s cell therapy and stem cell transplantation together with associated techniques opened a new insight into HIV pathology and gave us a glimpse of hope to find an effective cure strategy for HIV (Table 1).

**Table 1:** Major development steps of the cell or stem cell based HIV therapy during the last decades

Year	Improvement	Ref
1981	Cell transfer without conditioning	[44]
1984	Syngeneic SCT	[45]
1988	SCT together with anti HIV medication (Suramin)	[46]
1989	Allogeneic SCT	[47]
1990	Use of anti-retrovirals during SCT	[48]
2001	Allogeneic SCT combined with gene therapy	[49]
2005	Cord blood for SCT	[50]
2007	SCT with a CCR5-d32 homozygous graft	[10]
2010	In vivo (animal) modification of hematopoietic stem cells using zing fingers	[51]
2013	Combined CCR5-d32 cord blood with haplo bride	[52]
2016	Generating multi-HIV-antigen specific T-cells	[24]

This review highlights the previous experiences of stem cell based treatment approaches for HIV and outlines possible developments based on our current knowledge.

## An introduction into stem cell therapy

After the pioneer work of Thomas et al. stem cell transplantation (SCT) has become a standard treatment in high risk leukemia and lymphomas and in some non-malignant diseases like aplastic anemia or thalassemia [3]. Two major concepts have to be distinguished: the autologous and the allogeneic SCT. In autologous setting, patients donate their own stem cells (after mobilization and apheresis) to get reinfused after high dose chemotherapy to treat their cancer (in the majority non-Hodgkin’s lymphoma). In allogeneic SCT a matching donor is required. The criteria of matching are based on the human leukocyte antigen (HLA) system and 5 gene loci (A, B, C, DRB, DRQ) with 2 allelic variants each (=10 alleles total) have to be considered. For related or unrelated SCT a match of at least 9 of 10 HLA characteristics are recommended, whereas transfer of stem cells from cord blood requires only a 4 of 6 HLA match.

In fact, in haploidentical SCT there is only a 5 of 10 HLA match, but the donor is chosen from 1st grade relatives and therefore this haplotype is much more related to the recipient as compared with an unrelated donor with only 5 of 10 HLA match. However, severe grafts vs. host reactions have to be managed in all kind of stem cell sources except the special case of syngeneic SCT between identical twins. Based on our current knowledge, the cure from hematological malignancies is mainly based on a graft versus leukemia effect (as part of the graft vs host effect) carried out by a small proportion of allo-reactive anti-leukemia T-cells derived from the graft.

## Brief history of stem cell transplantation in patients with HIV

**Fig. 1:**
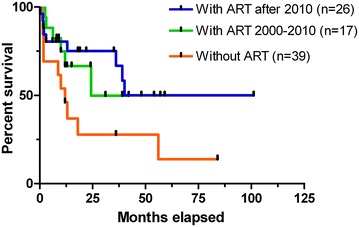
Clinical outcome of allogeneic stem cell transplantation (syngeneic, unrelated, related, haploidentical, and cord blood) in the pre-ART era and during the period form 2000–2010 and after 2010

The first stem cell transplantations in HIV+ patients have been performed unaware of the virus infection in the earliest 1980s. After these initial cases numerous HIV+ patients with concomitant malignancy or other hematological diseases received allogeneic SCT all over the world [4]. The outcome of this procedure was substantially improved after ART became a standard treatment for the recipients (Fig. 1). However, analysis of retrospective data of 111 transplantation of HIV+ patients indicated a slightly lower overall survival rate as compared to a matched control group of HIV negative patients. The reason for this observation is not fully understood but the differing pathogenesis of HIV-associated lymphomas may attribute to this outcome [5].

In the past, the major problem of patients entering into the state of AIDS is the immunodeficiency due to the loss of functional T-cells. In these early days and before the availability of efficient antiretroviral treatment options, some physicians tried to replace the missing lymphocytes by adoptive cell transfusions. Immunologically this is quite easy in a syngeneic setting and Lane et al. reported on HIV+ patients with a matching identical twin where T- and stem cell infusion were given to support the disturbed immune system [6]. In fact, this approach worked to increase the number of circulating T-cells but later on the transfused cells were infected by the uncontrolled virus and finally get eliminated by the cythopatic effect of HIV. A few years later, after effective antiretroviral medication was available, these approaches have been repeated with syngeneic donor lymphocyte transfusion ongoing ART but without sustained effect on the persistence of the virus [7].

Certainly, donor lymphocyte infusion may balance the lack of lymphocytes in these patients for a short period of time but even if these infusions have been given repetitively they did not reduce the number of latent infected cell. In this context, SCT should be much more efficient because pluripotent stem cells can replace T-lymphocytes continuously and the unavoidable graft vs. host effect may purge the reservoir from the old-infected cells and replace them with new, healthy cells. This strategy of “kill and replacement” works well in hematological diseases but failed in HIV infection. In all available reports where ART was discontinued after regular SCT the patients rebounded with the virus even in cases where years after SCT no traces of HIV were detectable as shown in the so call “Boston patients” [8]. These results are suggestive to conclude that a proposed graft versus HIV effect (as variation of the well described graft vs. leukemia effect) might not be powerful enough to clear all sanctuary sides where the virus may hide.

If SCT alone is not effective to eliminate the virus a possible alternative could be to make the transplanted cells resistant against reinfection. Several attempts by gene therapy techniques have been undertaken but the most compelling proof of principal was made in the “Berlin patient”. In this transplantation stem cells were used form a donor naturally lacking the essential HIV entry receptor CCR5 (CCR5-delta32 deletion) [9, 10]. After this encouraging case stem cell transplantation moved into the center of attention in terms of eradication and HIV cure [11].

Patients with HIV have an increased risk in developing cancer in comparison to non infected people. The incidence of AIDS defining malignancies has dramatically decreased after the introduction of ART as a standard treatment option. However, the incidence of Hodgkin’s lymphoma and non-Hodgkin’s lymphoma is still higher in the HIV+ population. High dose chemotherapy with autologous SCT has become a standard procedure in patients with aggressive or refractory lymphoma.

Today, we can look back on a larger number of patients infected with HIV receiving autologous SCT during their treatment of different types of lymphoma (Table 2). In comparison to HIV-negative patients receiving the same procedure there was no evidence of increased treatment related morbidity or mortality for HIV+ patients and the outcome in non-Hodgkin’s lymphoma is comparable [12].

**Table 2:** Use of autologous stem cell transplantation after high dose chemotherapy in different entities of non-Hodgkin´s lymphoma

No. patients	Entity	Results	Reference
20	NHL	85 % progression free survival	[53]
53	NHL, HL	Randomized with HIV-negative group: no significant difference in OS, PFS	[12]
68	NHL, HL	PFS 56 % at median follow up of 32 months	[54]
7	NHL	SCT with anti CD20 antibody	[55]
3	MM	CR	[56]
1	CNS-NHL	CR	[55]

Chemotherapy alone or in combination with irradiation is used routinely during cancer treatment as well as autologous and allogeneic stem cell transplantation. The myelosuppressive effect of chemotherapy is sustained but usually only temporary. Some chemotherapeutics (e.g., fludarabine) cause more cell line-specific and long-lasting effects. However, experiences with HIV+ patients receiving high dose chemotherapy and autologous stem cell transplantation have revealed a lack of sustained effect on the size of the viral reservoir [13].

In the past there were few reports indicating a possible curative effect of the autologous SCT on the course of HIV infection. Despite these casuistic cases there is much more evidence that autologous SCT may reduced the size of the reservoir but is not sufficient to eliminate the virus. Most recently, Zanussi et al. could show that a smaller amount of the residual provirus in the autologous graft is associated with the later burden of HIV-provirus in the peripheral blood [14].

This reduction of the reservoir size could be essential in strategies to purge infected cells from the provirus as demonstrated for the first time using the tre-recombinase. This enzyme is able to selectively cut off the integrated virus copy from the human genome. Consequently one strategy could consist in treating patients with high dose chemotherapy to minimize the immobile reservoir and then replacing the bone marrow with autologous cell, previously purged and provirus-free after in vitro preparation with the tre-recombinase. This procedure may have to be repeated two or three times but in the end a sustained elimination of the residual pool of infected cells could be achievable [15].

Another technique in removing the provirus from the host is the zinc finger based endonuclease (ZFN). ZFNs targeting sequences in HIV protease (ZFN1) reverse transcriptase (ZFN2 & 3), or integrase ZFN4. First results of preclinical testing showed a robust antiretroviral effect of these constructs. However, recently the first report of ZFN resistance mutation was reported. This single amino acid mutation within the thumb domain of reverse transcriptase conferred resistance to the activity HIV *pol*-specific ZNFs indicating a potential limitation of this approach [16].

## Possible interactions during SCT in HIV+ patients

It is generally consensus that ART has not to be discontinued during high dose chemotherapy and autologous and allogeneic SCT. However, patients receive several medications during the treatment which could interact with the antiretroviral therapy. For nucleoside reverse transcriptase inhibitors (NRTIs), the potential for direct drug–drug inactions is negligible because of the elimination route aside the CYP450 system but may have an indirect influence on the transporter-mediated renal clearance of other medications. More critical are protease inhibitors (PIs), non-NRTIs, and chemokine receptor antagonists, are extensively metabolized by CYP450 system and could interference with numerous anti-cancer agents [17]. One alternative could be the use of integrase inhibitors such like elvitegravir which is metabolized via CYP3A4 and not the CYP450 system.

A second concern in cancer treatment and ART medication is the possible temporary incapability of the patients to take oral medication. This can be caused by refractory emesis or sever mucositis. Anti-retrovirals are commonly not available for intravenous administration and therefor a longer period of ART discontinuation could lead to viral rebound and escape from intended therapeutic stem cell strategies. This dilemma could be solved by using parenteral anti-retrovirals like subcutaneous enfuvirtide which has been already tested in patients undergoing allogeneic SCT [18].

Anti thymocytic antibodies (ATG) used during allogeneic SCT contain functional antibodies against T cell markers including CD4, CCR5 and CXCR4 resulting in a depletion of HIV-1 target cells. Recently, it has been demonstrated that ATG causes a long-lasting reduction of CD4+ T cells in HIV+ patients receiving HAART after renal transplant. The CD4+ T cell recovery time varied widely from patient to patient; time to achieve CD4+ >200 cells/μL averaged 342 days and ranged from 18 days to over 2 years, despite maintenance on effective HAART therapy [19].

To our knowledge data concerning the influence of ATG on HIV-1 replication are not available. However, it can be suggested that ATG is capable of reducing the viral reservoir by depletion of infected CD4+ T cells and non-infected target cells during the period of application.

Cyclosporin A (CsA) is a potent immunosuppressive drug commonly used in the clinical setting for immunosuppression after allogeneic transplantation. CsA has been shown to inhibit T cell activation through a mechanism well defined at the molecular level. Briefly, signaling through the T cell receptor (TCR) induces an increase of intracellular Ca^2+^ that activates the phosphatase calcineurin, which dephosphorylates a number of transcription factors, including nuclear factor of activated cells (NFAT) and NF-ΚB. Since these proteins are essential activators of HIV-1 transcription, CsA is able to inhibit viral replication.

The rationale for the use of CsA during primary HIV infection was to decrease the heightened state of T cell activation observed in order to limit infection, and thus depletion, of CD4+ T lymphocytes, contributing to a better long-term preservation. Although an independent study suggested caution in the use of CsA in early HIV disease, CsA has successfully been used in vivo as an adjunct to HAART in patients showing an early increment of both percentage and absolute number of CD4+ T lymphocytes as compared to HAART-treated patients alone.

CsA was tested during primary HIV-infection: CsA was not detrimental to virus-specific CD8+ or CD4+ T cell responses. At week 48, the proportion of IFN-γ-secreting CD4+ and CD4+CCR7– T cells was significantly higher in the CsA+ HAART cohort than in the cohort, where HAART had been given exclusively. The authors suggested that rapid shutdown of T cell activation in the early phases of primary HIV-1 infection can have long-term beneficial effects and establish a more favorable immunologic set-point. Appropriate, immune-based therapeutic interventions may represent a valuable complement to HAART for treating HIV infection [20].

## Adoptive cell transfer

As figured out, T-cell transfusion have been considered and performed in syngeneic settings in several patients with HIV before. In comparison to this undirected approach the adoptive cell transfer is based on special characteristics of the selected cell source. Examples for this technique are the transplantation allogeneic dendritic cells or autologous modified T-cells carrying a chimeric antigen receptor (CAR) [21].

The first approaches of T-lymphocyte infusions were only casuistic—as described above. Another approach is the use of dendritic cell transfer that could be helpful in strategies to reduce the reservoir size. In a recent study, HIV+ patients where randomized to receive ether autologous dendritic cells which were pulsed with virus or cells which were not pulsed. In the treatment group a significant improvement of the size of the reservoir an a delayed replenishment after ART discontinuation was seen [22].

The third approach, so called CAR T-cells, may act as allo-reactive and high specific cytotoxic killer cells and experiences in patients with HIV+ have shown that these modified cells may persists over several years in the patients which could be essential to design CAR T-cells with the ability to control or reduce the HIV reservoir [23].

Recently, another progress in adoptive cell transfer was reported from Patel and coworkers. In this approach T cells form HIV antigen negative donors where ex vivo primed against HIV antigens and expanded resulting in a functional, multi-HIV specific cell line. In the presence of HIV infected cells these artificially primed cells released IFN-γ as a response to the antigen [24].

## HIV resistant cells

During the search for an appropriate animal model to study HIV infection in the 1980s researcher recognized a natural resistance in nonhuman primate species. The principal idea was to translate this resistance into patients with HIV by transplanting animal stem cells into humans which is technically feasible [25]. To achieve this several considerable difficulties have to be overcome: (1) the animal graft can not replace all functions of the human bone marrow driven cells, (2) graft versus host may become a major problem, (3) zoonosis have to be considered.

However, in 1996 a single patient received a graft from an adult baboon after non-myeloablative conditioning. The transplant resulted in an engraftment of a mixed chimerism of the baboon cell source. The viral load decreased during transplantation procedure from approximately 70,000–2100 cps/ml on day +4 but increased during engraftment and was stable the following 7.5 years at 45,000 cps/ml. Transplantation side effects were moderate and the patient developed no HIV-related complication although he was consistently without antiretroviral medication [26]. Probably because of the improvements of antiviral treatment, no further attempts in this direction were undertaken.

In 2009, Hütter and colleagues described successful hematopoietic stem cell transplantation in an HIV-1 infected patient by transferring CCR5-delta32 donor derived cells that harbour a natural resistance against HIV infection. These hematopoietic stem cells engrafted, proliferated and differentiated into mature myeloid and lymphoid cells. At present the patient is more than 9 years post allogeneic transplantation without the requirement of any antiretroviral treatment. Analysing peripheral blood cells and different tissue samples including gut, liver, and brain, no viral RNA load or proviral DNA could be detected [9, 27].

Limitations of this approach may appear by viral escape from the CCR5 based entry mechanism. During transmission of HIV, CCR5 is the preferred co-receptor for cell entry. However, during the time course of HIV infection, the virus is able to change its tropism to other chemokine co-receptors, such as CXCR4, and the role of CCR5 in maintaining HIV infection is still unclear. The “Berlin patient” harbored a CXCR4 tropic variant (X4) before transplantation that did not emerge after discontinuation of antiretroviral therapy, a phenomenon that is discussed by Symons et al. and cannot conclusively be explained. According to Symons the rebuild particles with CXCR4 tropic sequences behaved in vivo more like a CCR5 tropic virus [28].

Repeating this approach failed in several attempts due to early treatment and disease related fatalities [29]. One patient survived the critical phase of transplantation and acute GvHD but developed a viral escape from HIV with a non-CCR5 using variant [30]. Although this case represents a draw back of the concept of viral control by HIV resistant cell sources, there were several important differences between this patient an the “berlin case” as figured out by Bodor et al. Particulary, that the antiretroviral medication was discontinued very early so that the virus had enough time to evade before a stable engraftment of CCR5-delta32 cells was achieved [31].

A third patient who received the same treatment with CCR5-negative allogeneic stem cells managed transplantation procedure, engraftment and episodes of GvHD but is continuously on antiretroviral medication, making it impossible to clarify whether eradication is achieved even if there is no trace of HIV detectable [32].

Currently gathering more information from HIV patients undergoing allogeneic SCT the “European Project to guide and investigate the potential for HIV cure by Stem Cell Transplantation” (EpiStem) was founded in 2015 by an amfAR grant to offer participating transplant-centers to submit patient’s data and samples to evaluate the effects of transplantation in HIV patients with and without CCR5 deficient stem sources (http://www.epistem-project.org/). The three major goals of this project are: (1) to achieve the largest cohort of allogeneic SCT recipients with HIV-infection; (2) to collect scientific information on viral reservoirs using multiple complementary quantifications, molecular and functional characterization of the virus; (3) to explore innovative approaches using these unique cases of extremely low viral reservoir that help to gather more information on viral reservoirs during allogeneic SCT.

Since the kick-off, EpiStem has generated a prospective observational cohort of cases of allogeneic SCT in HIV-1-infected individuals. More than 15 patients have been included already and so far 3 patients have successfully passed the 6 months follow-up after transplantation while 6 patients have died shortly after transplantation. Preliminary analysis of virological and immunological data from blood and tissue samples shows a systematic reduction of HIV-1 reservoirs to very low levels after transplantation [33].

## Manufacturing of resistant cells

Based on work of Dorothee van Laer anti HIV gene therapy is considered to be classified into three groups: Class I includes genes as target structures that inhibit early stages of viral replication including invasion and integration. Targets of interest are the chemokine receptors as well as the reverse transcriptase and integrase. Class II gene therapy aims on the expression of HIV proteins during replication like transdominant negative mutants of Rev (tdRev) and ribozymes. They can suppress the cytopathic effect of the virus with the potential risk that transfected cells harboring the provirus may accumulate of a period of time. Thirdly, class III gene therapeutics inhibit the building or the release of the virus, thereby preventing new infection of other cells [34]. Based on a mathematical model to predict the possible impact of each class of HIV gene therapy on latency and replication, only class I gene therapy manipulated cells will gain prevalence and ultimately are able to control the infection [35].

Kang and co-workers performed the first allogeneic SCT in patients with HIV using genetically modified allogeneic stem cells. They transduced donor CD34+ cells with tdRev, engineered to inhibit viral replication through blocking of wild type Rev, a key HIV regulatory protein. This trial was partially successful as stable transfection of TdRev was measurable at least 2 years after allogeneic transplantation. The effect on HIV course was not clearly evaluable because administration of antiretroviral medication has not been interrupted [36].

Another approach is the use of small interfering RNA (siRNA). Although siRNA mediates sequence-specific RNA cleavage, expression of a single siRNA species frequently led to the emergence of HIV escape variants. Lo and co-workers reported a promising strategy of using polymerase (pol) II promoters to drive the expression of siRNAs against HIV-1. Both, the vector copy number and the promoter strength, directly affected the ability of the siRNA to inhibit HIV-1 replication. A combination of pol II and pol III promoters can express two different siRNAs to increase the efficacy against HIV-1 replication [37].

Clinical experiences have been collected using OZ1, a *tat/vpr* specific anti-HIV ribozyme, transfected with a retroviral vector. Mitsuyasu and co-workers administered an infusion of CD34+ cells transduced with OZ1 or treated with placebo to 74 HIV-1-infected adults. Over a period of 100 weeks, antiretroviral therapy was interrupted twice to provide positive selective pressure for the progeny of OZ1-transduced CD34+ cells. For the first time this study provided that cell-delivered gene transfer can significantly reduce HIV viral load [38].

During the last decade, Sangamo a biotech company located in Richmond, CA, USA, developed a specific ZFN which induces a double strand break in the CCR5 gene, leading to a none functional receptor and thereby mimicking the natural occurring CCR5-delta32 deletion. However, a complete suppression of all CCR5 expression on the cell surface is not feasible with any currently used gene therapy technique. In several phase I trials the dynamics and the outcome of ZFN manipulated cells were tested. HIV-1 infected patients donate their autologous T-lymphocytes which were expanded and transduced by the adenovirus vector and then re-infused. All patients had an increment in terms of HIV infection critical CD4+ cell count indicating an improvement of the immune state. Furthermore, these transduced T-cells expanded and reseeded the immunological important sides like the gut as tested in several biopsies. In a second experimental setting, the antiretroviral medication was discontinued after re-infusion of ZFN manipulated cells to increase the selective pressure and to enhance the expansion of artificial HIV resistant cells. As expected, all patients had a quick and reasonable rebound of the virus but interestingly in those patients found heterozygous for the CCR5-delta32 deletion some displayed signs of a real virus control [39]. In a third phase the additional use of the chemotherapeutic drug cyclophosphamide (CTX) was tested. Cyclophosphamide has only a moderate myelosuppressive effect but a strong immunosuppressive impact which could improve the ability of transplanted HIV-resistant cells to expand. After discontinuation of ART all three candidates rebounded 30–90 days later, in comparison to 2 week in probands receiving only the edited cells without CTX, indicating a longer virus control. However, only one patient in the CTX group developed a spontaneous virus control at the end of the observation period [40].

The second promising approach is featured by Calimmune Inc., LA, USA which uses a shRNA lenti-viral construct to down regulate CCR5 in combination with a small peptide linker C46 (together called Cal-1) which interacts with the fusion of the virus and the target cell surface. The combination of two independent mechanism might be more sufficient to achieve a higher grade of entry barrier. Cal-1 was already successfully tested in animal model where modified cells were permanent detectable in not infected test animal in the peripheral blood and in HIV-relevant tissue sites such as the gastrointestinal tract. Positive selection for gene-marked cells was also observed in blood and tissues following artificial infection, leading to maintenance of peripheral blood CD4+ T-cell counts in a normal range [41]. Calimmune is recruiting for a phase I study (NCT01734850) to test the safety and feasibility of the Cal-1 approach in a small cohort of HIV+ patients.

## Limitations of gene therapy

The conditions of HIV gene therapy have been calculated in mathematical models. Based on linear model of infection gene therapy strategies may control infection must have a higher proliferation rate or a longer lifespan compared to not edited cells or, alternatively, have beneficial effects that booster the cell count like immune effector function [42]. Furthermore, mathematic model clearly prefer modified hematopoietic stem cells rather peripheral cells to achieve a sufficient amount of edited cells to control the infection in a realistic period of time (in this case 10 years projection) [35]. In fact, this postulation from a theoretical point of view has already been come to practice as has been shown from Peterson et al. in an animal model, that after adaption of the clonal tracking method CCR5 gene-edited hematopoietic stem cells are capable of long-term engraftment and multiline age repopulation after autologous transplantation [41]. Finally, due to the high mutation rate of the virus, HIV may emerge from inhibitory gene therapy strategies as been shown by Berkhout et al. where in vitro cultures of HIV treated with siRNA contained nucleotide substitutions or deletions in or near the targeted sequence leading to resistance against the manipulation [43].

## Outlook

Autologous and allogeneic SCT in HIV+ patients is not even longer only relevant for hematologist. Conditioning regimen, immunological effects of the graft, and manipulation of cell sources may influence the course of HIV infection profoundly. However, our knowledge on the exact mechanism of SCT on the circulating infected cell, the resting reservoir cells or sanctuary sides for example in the brain are poorly understood and up to date not investigated in depth. SCT procedure has become a standard treatment option for HIV+ patients and does not track very much attention to the performing physicians. This routine could hinder the collection of specific data and findings to clarify still open questions in terms of latency and eradication.

**Fig. 2:**
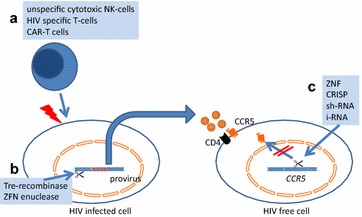
Current mechanism of cell based HIV therapy: **a** T-cell cytotoxicity by un-manipulated or manipulated cell sources. **b** Purging the reservoir from the HIV provirus. **c** Conferring HIV resistance by disruption of critical genes, e.g. CCR5 receptor

Currently, there is a run towards cell based therapy with adoptive cell transfer (not only in the field of HIV) (Fig. 2). Although these techniques are fascinating and promising, these attempts are at an early level with few patients and short period of follow-up so that there might be a risk in overestimating the potential of these approaches.

However, the first documented HIV cure is based on a stem cell approach and there is reasonable hope that this unique case will not stand alone in the future.
